# AlphaPulldown2—a general pipeline for high-throughput structural modeling

**DOI:** 10.1093/bioinformatics/btaf115

**Published:** 2025-03-14

**Authors:** Dmitry Molodenskiy, Valentin J Maurer, Dingquan Yu, Grzegorz Chojnowski, Stefan Bienert, Gerardo Tauriello, Konstantin Gilep, Torsten Schwede, Jan Kosinski

**Affiliations:** European Molecular Biology Laboratory Hamburg, Hamburg 22607, Germany; Centre for Structural Systems Biology (CSSB), Hamburg 22607, Germany; European Molecular Biology Laboratory Hamburg, Hamburg 22607, Germany; Centre for Structural Systems Biology (CSSB), Hamburg 22607, Germany; European Molecular Biology Laboratory Hamburg, Hamburg 22607, Germany; Centre for Structural Systems Biology (CSSB), Hamburg 22607, Germany; European Molecular Biology Laboratory Hamburg, Hamburg 22607, Germany; Biozentrum, University of Basel, Basel 4056, Switzerland; Computational Structural Biology, SIB Swiss Institute of Bioinformatics, Basel 4056, Switzerland; Biozentrum, University of Basel, Basel 4056, Switzerland; Computational Structural Biology, SIB Swiss Institute of Bioinformatics, Basel 4056, Switzerland; European Molecular Biology Laboratory Hamburg, Hamburg 22607, Germany; Centre for Structural Systems Biology (CSSB), Hamburg 22607, Germany; Biozentrum, University of Basel, Basel 4056, Switzerland; Computational Structural Biology, SIB Swiss Institute of Bioinformatics, Basel 4056, Switzerland; European Molecular Biology Laboratory Hamburg, Hamburg 22607, Germany; Centre for Structural Systems Biology (CSSB), Hamburg 22607, Germany; Molecular Systems Biology Unit, European Molecular Biology Laboratory, Heidelberg 69117, Germany

## Abstract

**Summary:**

AlphaPulldown2 streamlines protein structural modeling by automating workflows, improving code adaptability, and optimizing data management for large-scale applications. It introduces an automated Snakemake pipeline, compressed data storage, support for additional modeling backends like UniFold and AlphaLink2, and a range of other improvements. These upgrades make AlphaPulldown2 a versatile platform for predicting both binary interactions and complex multi-unit assemblies.

**Availability and implementation:**

*AlphaPulldown2* is freely available at https://github.com/KosinskiLab/AlphaPulldown.

## 1 Introduction

Recent advancements in Artificial Intelligence (AI)-based structural prediction, driven by tools such as AlphaFold2 (Jumper *et al.* 2021), RoseTTAFold (Baek *et al.* 2021), and ColabFold (Mirdita *et al.* 2022), have remarkably improved our capacity to predict protein–protein interactions (PPIs) and the architecture of protein complexes. The associated confidence scores can be also used to predict whether two proteins would interact, facilitating high-throughput computational screens of PPIs (Humphreys *et al.* 2021, Burke *et al.* 2023, Yu *et al.* 2023, Razew *et al.* 2024).

We previously introduced the AlphaPulldown Python package (Yu *et al.* 2023) to streamline PPI screens and facilitate high-throughput modeling of higher-order complexes using AlphaFold-Multimer (Evans *et al.* 2021). AlphaPulldown separates the AlphaFold2 pipeline into CPU-based calculation of input features [multiple sequence alignments (MSAs) and templates] and GPU-based structure prediction, reducing computational time. It offers four modes: pulldown, all-versus-all, homo-oligomer, and custom (Supplementary Fig. S1). The pulldown mode screens interactions between one or more “bait” proteins and a list of candidates, mimicking pulldown assays. The all-versus-all mode automatically predicts pairwise PPIs between all proteins in a provided list, useful for interaction network prediction. The homo-oligomer mode facilitates the modeling of alternative oligomeric states. The custom mode allows flexible input combinations of proteins or fragments. AlphaPulldown reuses input features pre-calculated for full-length proteins when modeling fragments, avoiding costly recalculation, and retains original residue numbering in the resulting models. It includes an integrated analysis pipeline that enriches native AlphaFold2 scores with additional evaluation metrics such as pDockQ (Bryant *et al.* 2022) and physical parameters of interfaces (Malhotra *et al.* 2021, Agirre *et al.* 2023), generating a graphical summary in a Jupyter notebook for comprehensive analysis of model confidence and interaction properties. AlphaPulldown has been utilized in a range of applications, demonstrating its effectiveness in PPI screens, modeling individual complexes, and interface scoring (Seidel *et al.* 2023, Bonchuk *et al.* 2024, Kadhim *et al.* 2024, Lapcik *et al.* 2024, Sellés-Baiget *et al.* 2025).

Despite its utility, challenges in automation, code adaptability, and management of the resulting models and data persisted. Moreover, new customized modeling protocols have emerged for adjusting modeling outcomes using modified MSAs, templates, or distance restraints (Mirabello *et al.* 2024, Stahl *et al.* 2024). In response, we introduce AlphaPulldown version 2.0, incorporating significant improvements in user experience, modeling capabilities, and available modeling and evaluation features. This updated version offers a comprehensive suite for modeling both binary PPIs and multi-unit assemblies, positioning it as a comprehensive toolbox for high-throughput protein structure prediction.

## 2 Software description

### 2.1 Usability improvements and software management

#### 2.1.1 Automation and new configuration syntax

**Figure 1.**
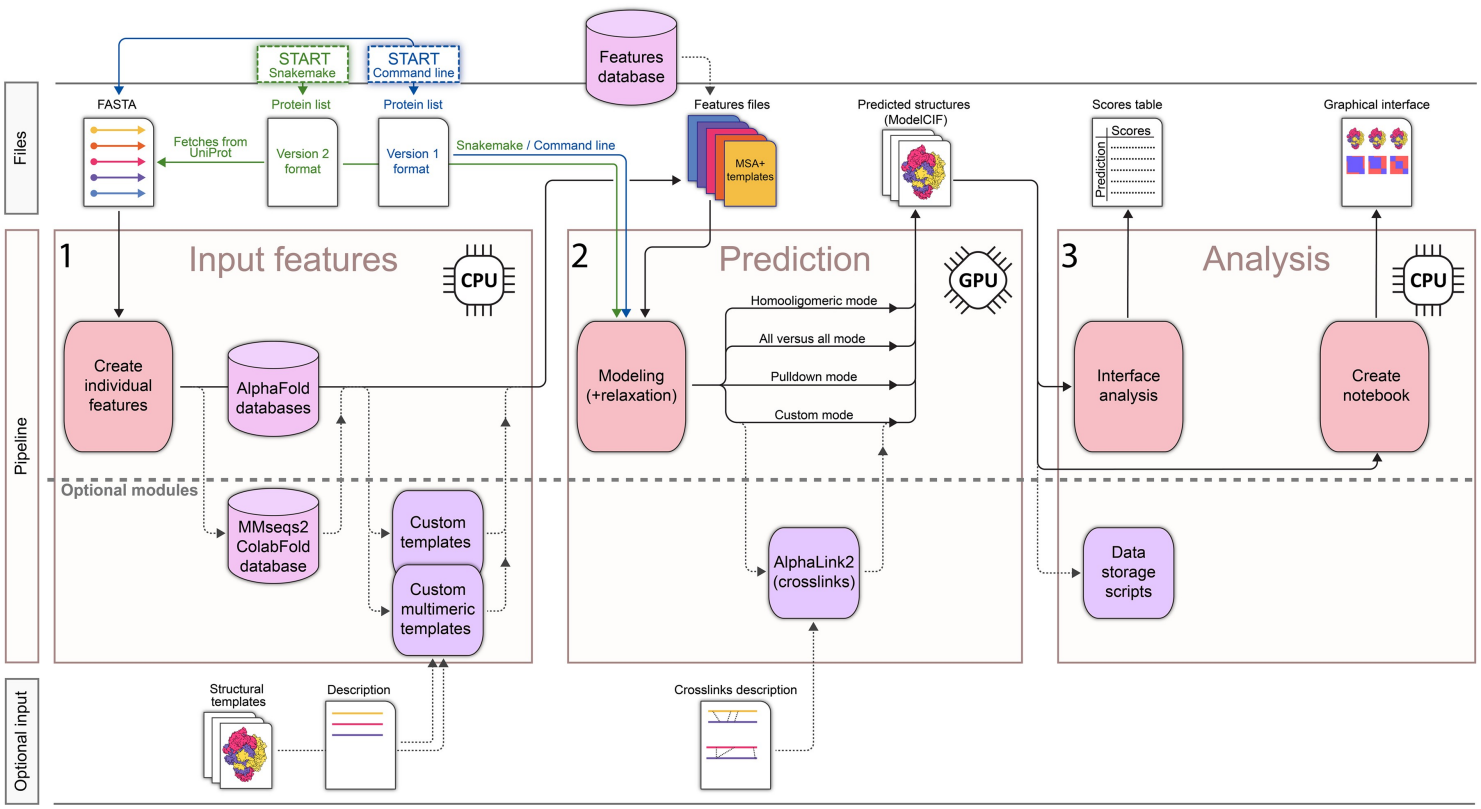
The workflow of structural modeling with AlphaPulldown2. For better allocation of resources, AlphaPulldown2 separates the AlphaFold2 pipeline into CPU-based calculation of input features and results analysis (1,3) and GPU-based structure prediction (2). The pipeline can use a new Version 2 input format that can define all Version 1 modes using simplified syntax. Additional input like cross-links and multimeric templates can be provided. The resulting models in ModelCIF format and confidence scores are summarized using an interactive, graphical interface. Features and output files are compressed to decrease storage requirements. The entire pipeline can be run automatically using Snakemake.

In large-scale structural modeling, managing multiple modeling tasks (jobs) and computational resources becomes a bottleneck. Thus, to further automate AI-based structural modeling, we developed an automated scalable and reproducible pipeline for AlphaPulldown2 using the Snakemake workflow management system (Mölder *et al.* 2021). This pipeline replicates the original AlphaPulldown workflow, but now runs all steps automatically based on an initial configuration (Fig. 1). Snakemake internally uses Singularity containers (Kurtzer *et al.* 2017) to ensure reproducibility and compatibility with various compute architectures, including cloud or local clusters. It reschedules jobs with settings adjusted based on failure reasons and resumes from saved checkpoints. Snakemake also simplifies the installation process, as AlphaPulldown is now available on DockerHub (Merkel 2014) and is automatically installed during the first execution.

We also introduced a new syntax for configuring the modeling jobs, which unifies all AlphaPulldown modes—pulldown, homo-oligomer, all-versus-all, and custom—into a single, flexible format (Supplementary Fig. S1). Within this format, AlphaPulldown2 additionally allows users to provide UniProt IDs of the input proteins (The UniProt Consortium 2023) and the sequences will be fetched automatically. This simplifies input preparation and allows users to define complex modeling scenarios with ease. While the original scripts and syntax remain available, the Snakemake pipeline and new configuration syntax enhance automation and usability.

#### 2.1.2 Storage management

High-throughput AI-based structure prediction requires extensive storage, as input features and output files with confidence scores can easily accumulate to terabytes of data. To mitigate this, we implemented several space-saving measures. Input features in PKL format (Python object serialization) are now compressed using the XZ format with the LZMA2 compression algorithm, reducing file sizes by 97.7% (55 GB instead of 2.4 TB for 20581 human proteins). Output PKL files are compressed too, and usually unnecessary data (aligned confidence probabilities, distograms, and masked MSAs) are removed before saving. This reduces the average size of output directories by 97%. These compressions are optional, with a script available for cleaning and compressing the data later. For instance, in the case of modeling 1000 random protein pairs, storage requirements dropped from 3.5 TB to 102 GB with AlphaPulldown2.

#### 2.1.3 Improved code organization and testing framework

To support community interest, feature requests, and our development goals, we have restructured the AlphaPulldown codebase. AlphaPulldown was initially built on AlphaFold-Multimer, but other implementations like OpenFold (Ahdritz *et al.* 2024) and UniFold (Li *et al.* 2022) have since emerged. UniFold, in particular, offers the capabilities of AlphaFold2 and includes code for training and fine-tuning AlphaFold2 weights. New tools such as AlphaFold3 (Abramson *et al.* 2024), OpenFold Multimer, and HelixFold3 (Liu *et al.* 2024) are also becoming available. To ensure that AlphaPulldown is able to timely incorporate these folding programs in the future, we reorganized the architecture of AlphaPulldown2 to allow flexible integration of various modeling backends. Input features can be generated through a unified pipeline using AlphaFold2 or MMSeqs2 (Steinegger and Söding 2017, Mirdita *et al.* 2022) and passed to the user’s chosen backend. For example, we added backend support for UniFold and AlphaLink2 (see Section 2.3), and additional backends can be integrated using this modular system.

Further improvements include code refactoring for clarity and efficiency, the removal of redundancies, and the addition of automated tests. We also implemented continuous integration and continuous delivery/deployment (CI/CD) pipelines to streamline ongoing development. AlphaPulldown docker images are automatically built and pushed to DockerHub upon each new software release to the AlphaPulldown GitHub repository, ensuring that the software remains up to date. Lastly, we expanded the usage manual to provide more comprehensive documentation.

### 2.2 Ensuring FAIR principles through ModelCIF support

In the era of ubiquitous structural modeling, one major challenge is ensuring that the resulting data are both reproducible and accessible in a standardized format. The lack of detailed metadata in the traditional PDB format limits the ability to reproduce results and assess model reliability.

To address this, we have added support for the ModelCIF format (Vallat *et al.* 2023). ModelCIF, an extension of the PDBx/mmCIF dictionary (Westbrook *et al.* 2022), is specifically designed to accommodate computationally generated models. ModelCIF aligns with the FAIR (Findable, Accessible, Interoperable, and Reusable) principles, enabling detailed documentation of the modeling process, including software versions, parameters, and information about used sequence and template databases. The global confidence scores as well as local pLDDT and pairwise alignment error matrices are stored in the files as well. This extension ensures that models predicted using AlphaPulldown2 can be stored with all necessary metadata for future replication and analysis. The introduction of ModelCIF support also ensures that AlphaPulldown2-generated models are fully compatible with repositories like ModelArchive (https://www.modelarchive.org/) (Schwede *et al.* 2009), facilitating model deposition. This integration not only facilitates compliance with FAIR principles but also provides a standardized and reliable way to store, share, and evaluate high-throughput structural modeling data.

### 2.3 Cross-link-driven modeling through the integration of AlphaLink2

Cross-linking mass spectrometry (XL-MS) is a powerful experimental technique used to study PPIs and macromolecular complexes (Graziadei and Rappsilber 2022). In XL-MS, chemical cross-linkers covalently bind specific amino acid residues that are in proximity. By analyzing these cross-linked peptides via mass spectrometry, the cross-linked residue pairs can be identified and used as distance restraints in structural modeling.

AlphaLink2 (Stahl *et al.* 2024) is a modified version of AlphaFold2, based on the UniFold implementation, that incorporates cross-linking data as modeling restraints within the AlphaFold2 neural network. This enhancement improves the accuracy of structural models, particularly for large protein complexes and challenging PPIs.

In AlphaPulldown2, we have implemented AlphaLink2 as an optional backend, enabling seamless integration of cross-link-driven modeling into the existing workflow. The process begins with feature generation using the standard AlphaFold2 or MMSeqs2 pipelines, after which these features are passed to AlphaLink2, where cross-linking restraints are applied to guide structure prediction. This integration allows users to perform both unconstrained and restraint-driven modeling within the same software framework, offering flexibility for various use cases while benefiting from the full range of features provided by AlphaPulldown2.

### 2.4 Extended modeling applications

AlphaPulldown2 offers a wide range of parameters and features that expand the capabilities of AlphaFold2. These include options for adjusting the number of recycles, specifying the number of output models, and selecting between different sequence search methods, such as the standard AlphaFold2 pipeline or the faster MMSeqs2. Users can also choose between different template search methods, like the faster HMMER (Finn *et al.* 2015) or the more accurate HHsearch (Steinegger *et al.* 2019). These customizable parameters enable users to optimize modeling protocols, whether prioritizing speed or accuracy.

AlphaPulldown2 also introduces enhanced functionalities for controlling predictions. Users can adjust the MSA by toggling the pairing of sequences from the same species or modifying MSA depth to increase the diversity of models (Monteiro da Silva *et al.* 2024). Additionally, custom templates, including multimeric templates, can be used. The multimeric templates allow users to impose the relative orientation of template chains on their models, improving the accuracy of complexes that cannot be accurately predicted by AlphaFold2 alone (Mirabello *et al.* 2024). This feature is particularly useful for refining pre-calculated models of smaller complexes or partially resolved experimental structures by adding missing regions or proteins. Additionally, it can automatically remove clashing residues and/or regions of low confidence (low pLDDT scores) when previous AlphaFold2 models are provided as templates. Since the reliance of AlphaFold2 on templates varies with the depth of the MSA, AlphaPulldown2 includes an automated mode that samples a gradient of MSA depths. This mode enables users to fine-tune the degree to which templates influence the final model.

Finally, the analysis pipeline has been enriched with the commonly used and requested average pLDDT and PAE scores at protein interfaces, offering a more comprehensive assessment of PPIs and model quality.

### 2.5 Repository of input features for model organisms

The generation of input MSA and template features is computationally intensive and often redundantly performed by different labs for the same proteins. This leads to unsustainable use of resources and time. Thus, we released a web-based repository (linked from the AlphaPulldown GitHub page) of the input features for the proteomes of 14 model organisms. Users can download individual features in compressed PKL format, allowing them to proceed directly to structure prediction without the need for MSA and template generation. This approach not only accelerates workflows but also reduces global computational costs and the associated energy consumption from repeatedly generating input features for the same proteins.

## 3 Conclusions, discussion, and future plans

AlphaPulldown2 represents a significant step forward in high-throughput AI-based structural modeling, offering a versatile and customizable platform for protein complex predictions. With its new automation features, support for different modeling backends, and the ability to integrate experimental data like cross-links, AlphaPulldown2 further enhanced the modeling quality. Its optimizations in storage management and computational efficiency also make it more sustainable for large-scale projects, reducing both time and resource consumption.

Although AlphaFold3 source code and its reproductions like HelixFold3 and Chai-1 have been released, they require further testing before they can be confidently incorporated into workflows such as AlphaPulldown. Additionally, the more restrictive licensing terms of these new programs may present challenges, particularly for high-throughput applications and for depositing models into public databases. For protein complex modeling, AlphaFold3 shows approximately 10% improvement in accuracy over AlphaFold2 (Abramson *et al.* 2024). However, AlphaFold3 has been shown to hallucinate false secondary structures more frequently than AlphaFold2 (Abramson *et al.* 2024). Thus, even though AlphaPulldown relies on AlphaFold2 and AlphaFold-Multimer, it remains a robust and reliable tool for high-throughput protein complex modeling.

We plan to incorporate additional modeling backends, including OpenFold. We are also working on expanding scoring functions and could integrate permissively licensed reproductions of AlphaFold3. Since the AlphaFold2 backend requires substantial computing time and memory (Supplementary Figs S2–S3), improving computational efficiency without compromising accuracy is another future direction. Although AlphaPulldown provides widely used and tested scores, their ability to distinguish true from false positive interactions has not yet been exhaustively tested, which should be addressed in future work. Thanks to the new modeling backends, future developments will also enable the incorporation of non-protein molecules and post-translational modifications.

## Supplementary Material

btaf115_Supplementary_Data
