# Supplementary material for: AlphaPulldown2—a general pipeline for high-throughput structural modeling
Source: Bioinformatics. 2025 Mar 14;41(3):btaf115. doi: 10.1093/bioinformatics/btaf115 (PMC11937959; doi:10.1093/bioinformatics/btaf115)
Supplement: btaf115_Supplementary_Data [file btaf115_supplementary_data.zip › AlphaPulldown2_SupplementaryInformation_revision_v3.pdf]

Supplementary information for “AlphaPulldown2 – a general pipeline for high-throughput structural modeling:

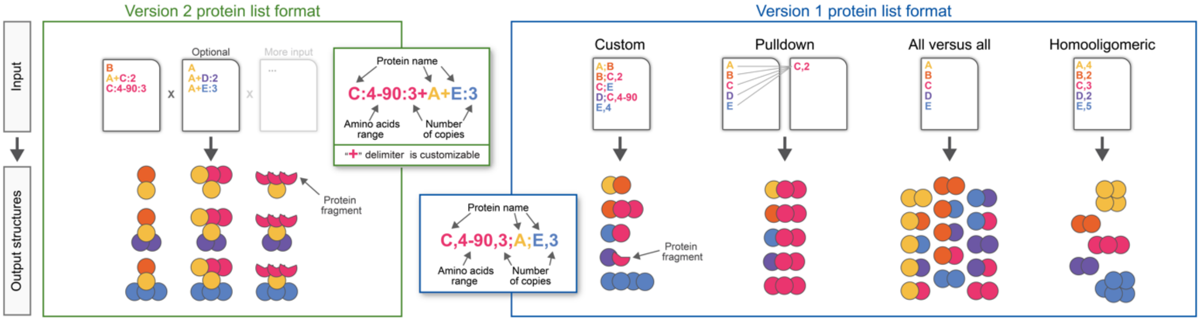

Supplementary Fig. 1. AlphaPulldown modeling modes and input syntax. Version 1 of AlphaPulldown (right) includes four distinct modeling modes: pulldown, all-versus-all, homo-oligomer, and custom, each requiring specific input syntax. In version 2 (left), these syntaxes are consolidated into a single, versatile format applicable to all modes. Both versions allow the selection of specific amino acid residue ranges for modeling, extracted from the full-length protein input, with the resulting models retaining the original residue numbering. AlphaPulldown2 supports both input formats to ensure backward compatibility.

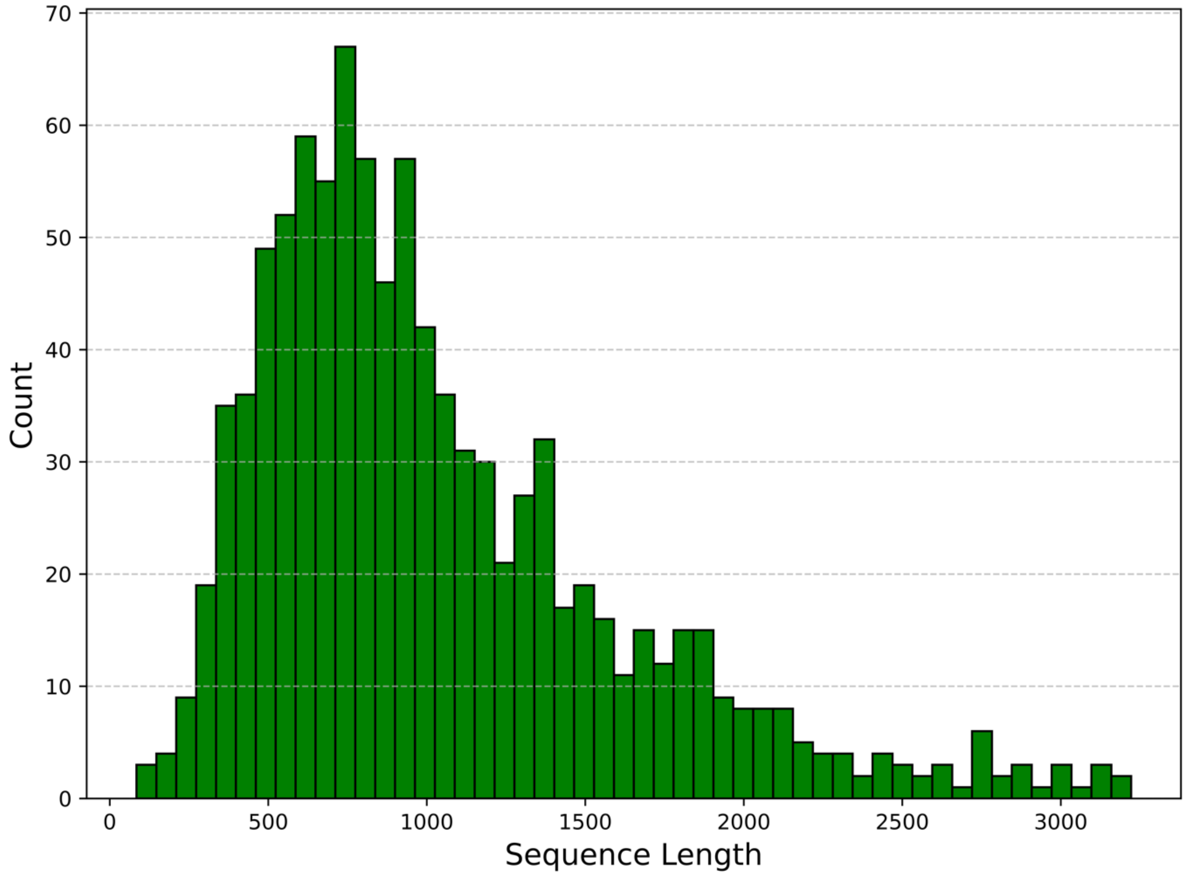

Supplementary Fig. 2. Sequence Length Distribution of Protein Dimers. Histogram depicting the length distribution of 1,000 randomly selected dimers from the human proteome.

(a)

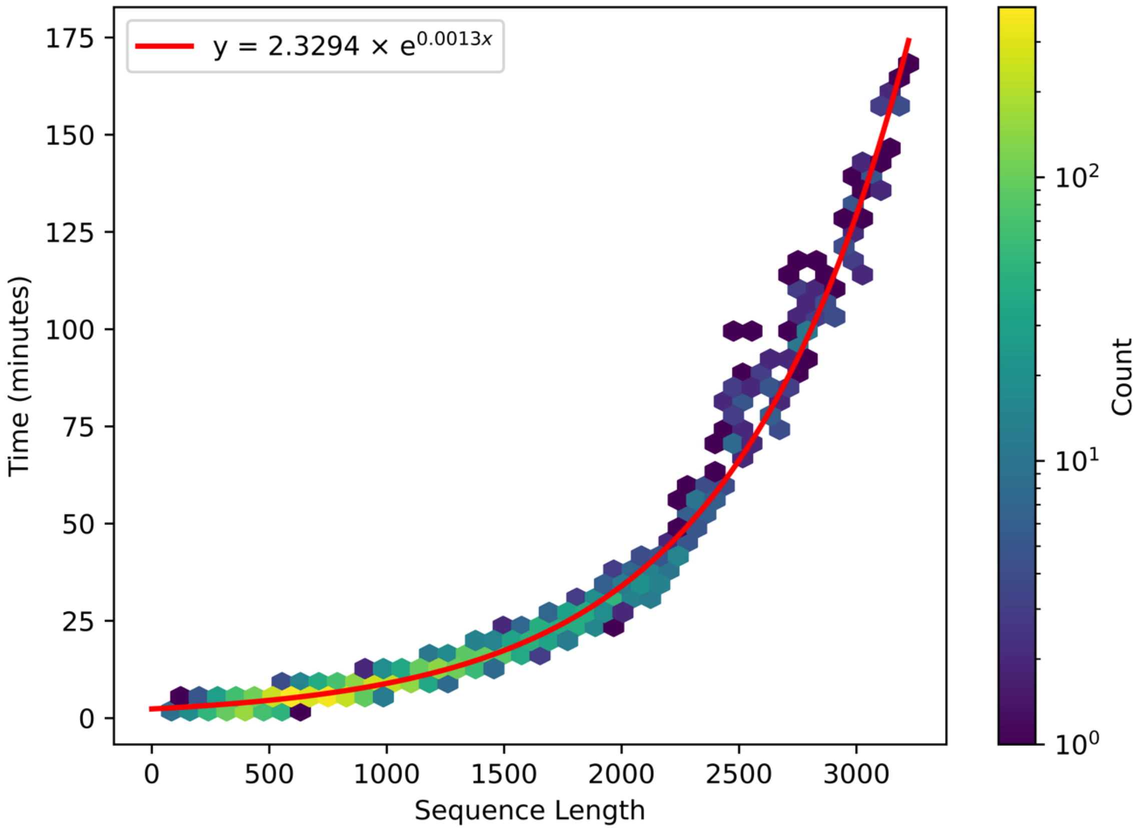

y = 2.3294 × e^0.0013x
Time (minutes)
Sequence Length
Count
0
25
50
75
100
125
150
175
0
500
1000
1500
2000
2500
3000
10^0
10^1
10^2

(b)

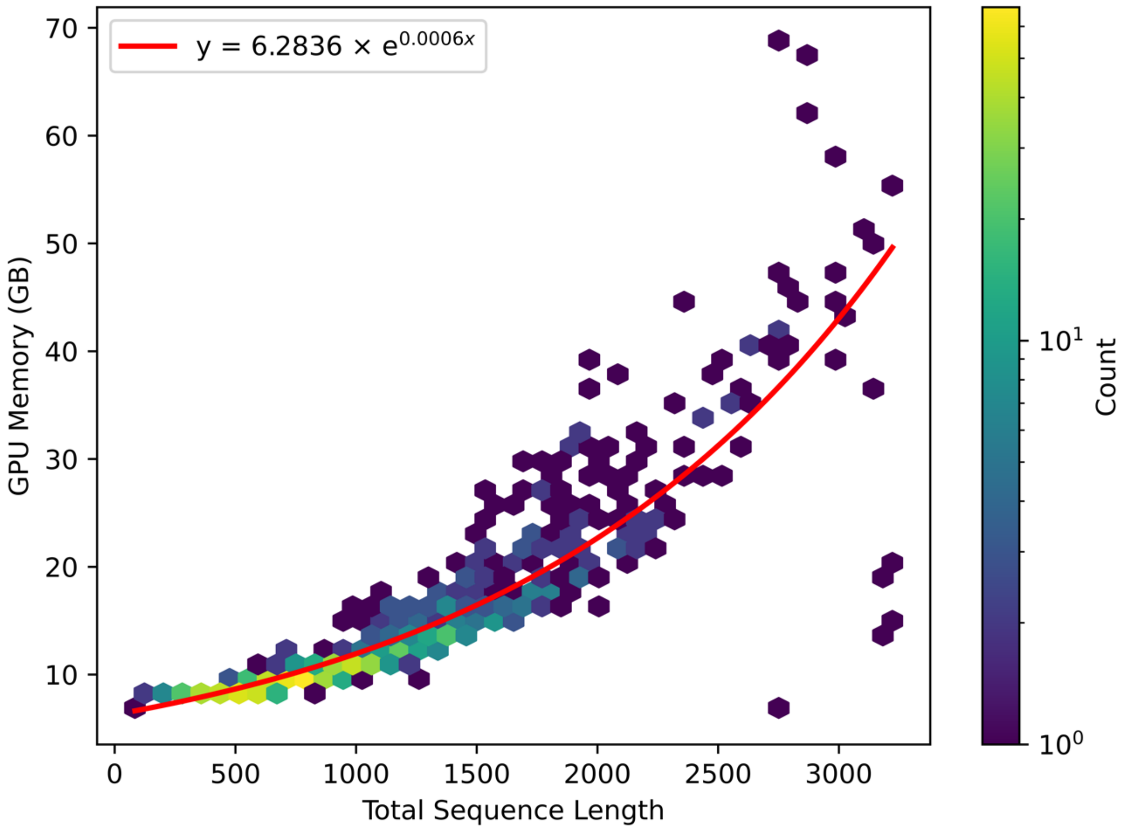

Supplementary Fig. 3. Computational Performance Benchmarks of AlphaPulldown on a NVIDIA RTX 3090 GPU. **(a) Prediction Time** for all five AlphaFold2 models as a function of the total length of protein dimers (one prediction per model). Presented as a hexbin plot with exponential fitting using the least squares method. **(b) Peak RAM Usage** as a function of the total length of protein dimers. Presented as a hexbin plot with exponential fitting.
