# Supplementary figures and images for: AlphaPulldown2—a general pipeline for high-throughput structural modeling
Source: Bioinformatics. 2025 Mar 14;41(3):btaf115. doi: 10.1093/bioinformatics/btaf115 (PMC11937959; doi:10.1093/bioinformatics/btaf115)

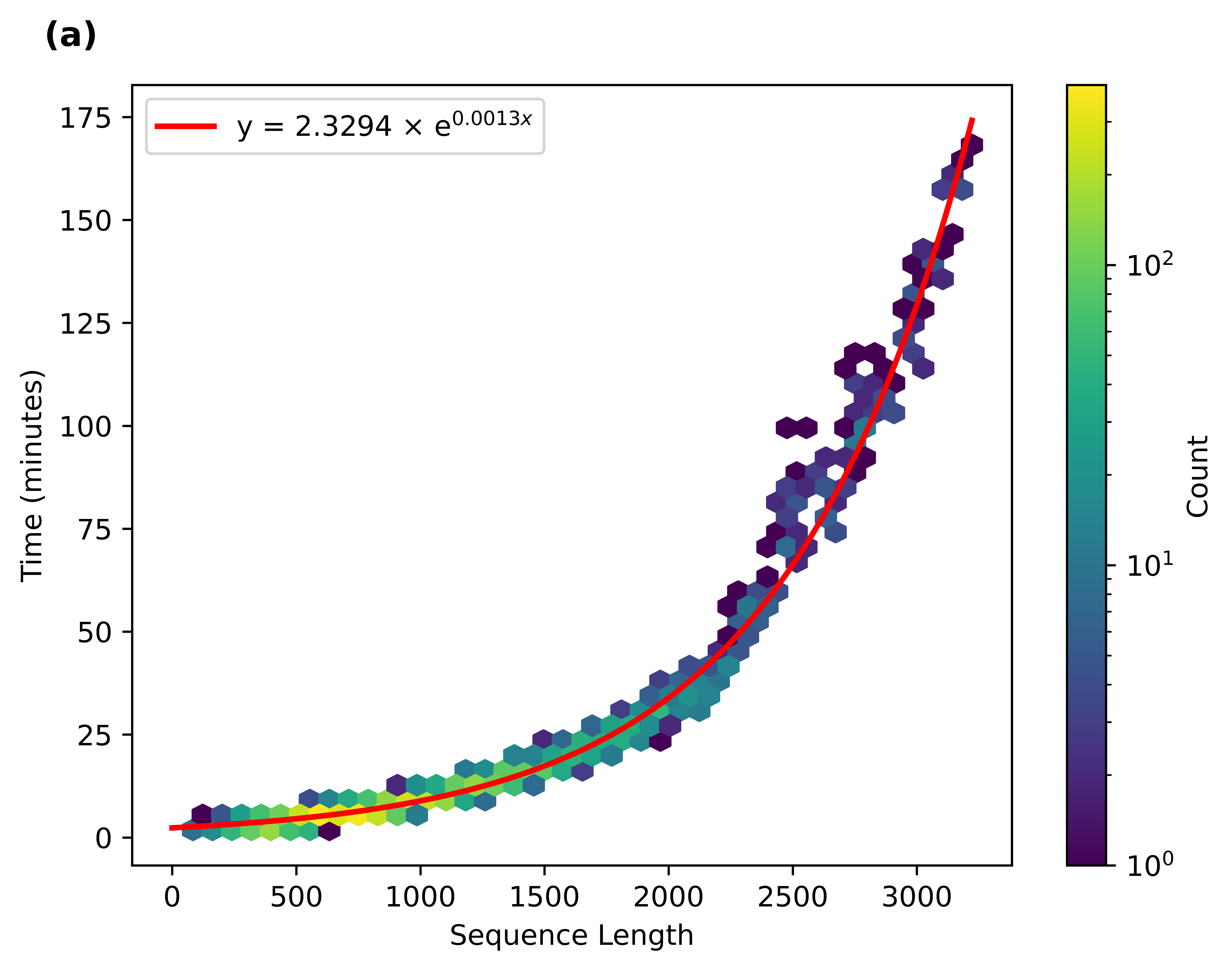

Supplement: btaf115_Supplementary_Data [file btaf115_supplementary_data.zip › Fig.S3a.high_resolution.png]

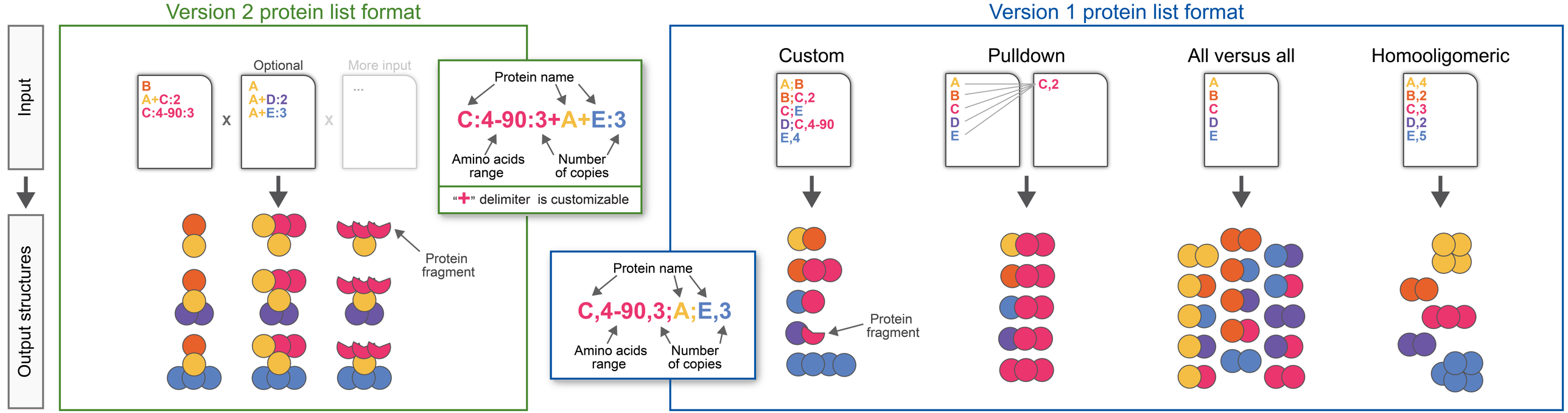

Supplement: btaf115_Supplementary_Data [file btaf115_supplementary_data.zip › Fig.S1.high_resolution.png]

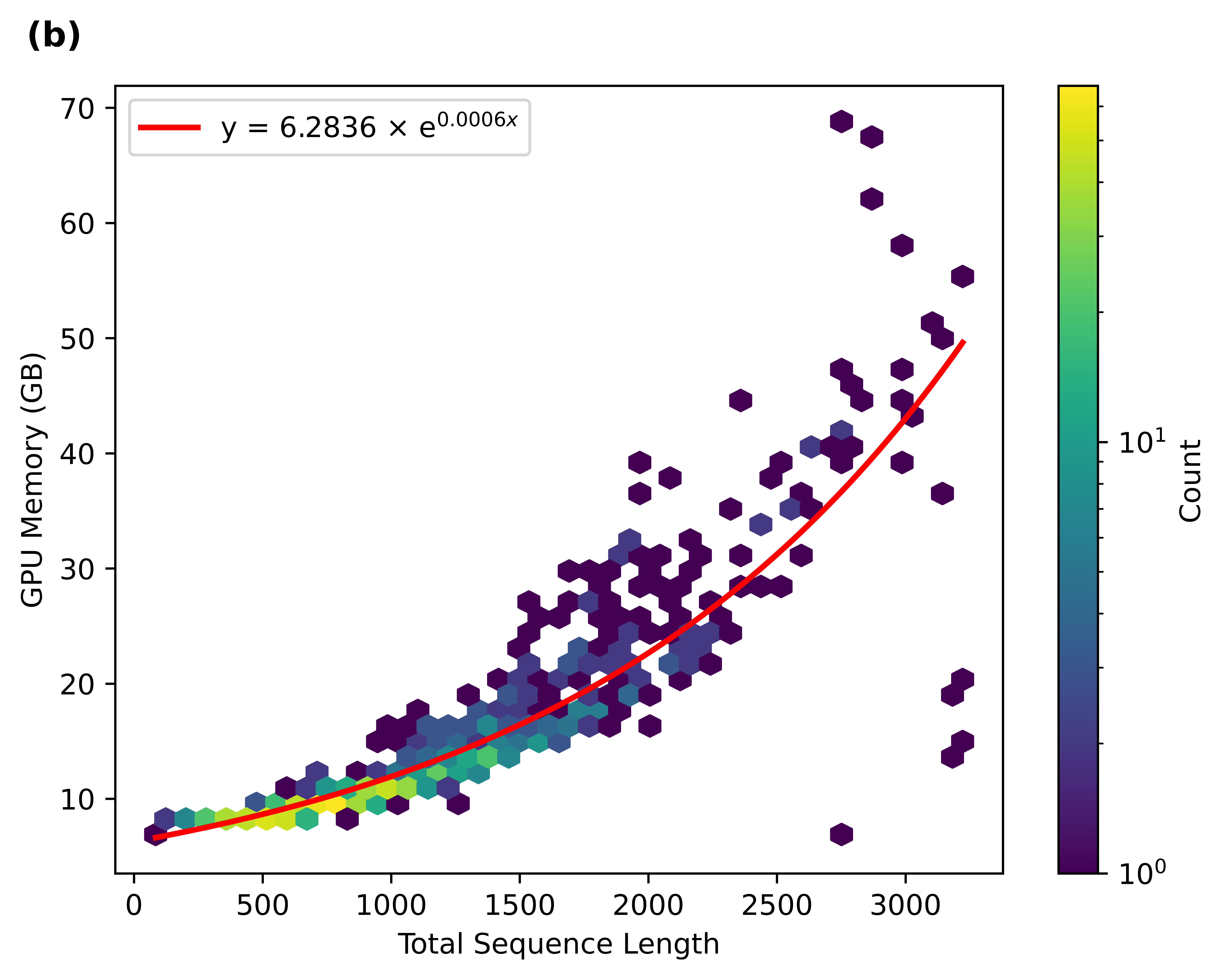

Supplement: btaf115_Supplementary_Data [file btaf115_supplementary_data.zip › Fig.S3b.high_resolution.png]
